# Impact of market-based home fortification with micronutrient powder on childhood anemia in Bangladesh: a modified stepped wedge design

**DOI:** 10.3389/fnut.2023.1271931

**Published:** 2024-01-05

**Authors:** Haribondhu Sarma, Mahfuzur Rahman, Md. Tariqujjaman, Mohammad Ashraful Islam, Mduduzi N. N. Mbuya, Grant J. Aaron, Sufia Askari, Catherine Harbour, Rudaba Khondker, Moniruzzaman Bipul, Sabiha Sultana, Mohammad Ashikur Rahman, Shaima Arzuman Shahin, Morseda Chowdhury, Kaosar Afsana, Samik Ghosh, Cathy Banwell, Catherine D’Este, Mihretab Salasibew, Lynnette M. Neufeld, Tahmeed Ahmed

**Affiliations:** ^1^National Centre for Epidemiology and Population Health, Australian National University, Acton, ACT, Australia; ^2^Nutrition Research Division, icddr,b, Dhaka, Bangladesh; ^3^Global Alliance for Improved Nutrition, Washington, DC, United States; ^4^Global Alliance for Improved Nutrition, Geneva, Switzerland; ^5^Maternal Child Health & Nutrition, Sight and Life, Geneva, Switzerland; ^6^The Children’s Investment Fund Foundation, London, United Kingdom; ^7^Global Alliance for Improved Nutrition, Dhaka, Bangladesh; ^8^Health, Nutrition and Population Program, BRAC, Dhaka, Bangladesh

**Keywords:** home fortification, micronutrient powder, market-based program, anemia, children, Bangladesh

## Abstract

**Background:**

Anemia poses a significant public health problem, affecting 1.6 billion people and contributing to the loss of 68.4 million disability-adjusted life years. We assessed the impact of a market-based home fortification program with micronutrient powder (MNP) called Pushtikona-5 implemented by Bangladesh Rural Advancement Committee (BRAC) on the prevalence of anemia among children aged 6–59 months in Bangladesh.

**Methods:**

We used a modified stepped wedged design and conducted three baseline, two midline, and three endline surveys to evaluate the Pushtikona-5 program implemented through three BRAC program platforms. We interviewed children’s caregivers, and collected finger-prick blood samples from children to measure hemoglobin concentration. We also collected data on coverage of Pushtikona-5 and infant and young child feeding (IYCF) practices. We performed bivariate and multivariable analysis and calculated adjusted risk ratios (ARRs) to assess the effect of program outcomes.

**Results:**

A total of 16,936 households were surveyed. The prevalence of anemia was 46.6% at baseline, dropping to 32.1% at midline and 31.2% at endline. These represented adjusted relative reductions of 34% at midline (RR 0.66, 95%CI 0.62 to 0.71, value of *p* <0.001) and 32% at endline (RR 0.68, 95%CI 0.64 to 0.71, value of *p* <0.001) relative to baseline. Regarding MNP coverage, at baseline, 43.5% of caregivers surveyed had heard about MNP; 24.3% of children had ever consumed food with MNP, and only 1.8% had consumed three or more sachets in the 7 days preceding the survey. These increased to 63.0, 36.9, and 4.6%, respectively, at midline and 90.6, 68.9, and 11.5%, respectively, at endline.

**Conclusion:**

These results show evidence of a reduction in the prevalence of anemia and an improvement in coverage. This study provides important evidence of the feasibility and potential for impact of linking market-based MNP distribution with IYCF promotion through community level health workers.

## Introduction

Anemia poses a significant public health problem, affecting 1.6 billion people and contributing to the loss of 68.4 million disability-adjusted life years (1). Childhood anemia is widespread across regions and countries. In South Asia (including Bangladesh and India), preschool children are the most affected. More than half of Bangladeshi children under age five (51%) are estimated to be anemic (2), and prevalence has consistently been high in the past several decades. Although multiple factors can result in anemia, iron deficiency is considered the most common cause (1). Anemia can have severe effects on children, including impaired cognitive, mental and motor development (3, 4) and several chronic diseases (5), and results in fatigue and reduced work productivity among adult women (6).

Multiple interventions are available to address childhood anemia. Home fortification of foods with micronutrient powders (MNPs), managed through the primary healthcare setting using community vendors, has the potential to reduce anemia and iron deficiency in children (5, 7). The World Health Organization (WHO) recommends the use of MNPs in areas where diverse foods are unavailable or unaffordable and where industrial fortification of food with multiple micronutrients is inadequate to fill nutrient gaps (8–10). Despite these recommendations, few countries have scaled up home fortification to a national level. A global assessment published in 2013 (11) found that only 12 out of 63 home fortification programs in low-income countries were scaled up nationally, the recent evidence specifically focusing on the effect of interventions distributing home fortification products (HFP) on infant and young child feeding (IYCF) practices instead of evaluating home fortification with MNP implementation at global scale (12).

Most of these nationally scaled-up programs were implemented with the support of external funding agencies and distributed MNPs to caregivers at no cost (11). This dependence on external funding creates concerns about the programs’ long-term sustainability (13). Market-based MNP promotion has been identified potentially as a sustainable approach to home fortification (14, 15) because it generates revenue to subsidize operational costs. Having local health workers sell MNPs to caregivers as part of the promotion of home fortification can help programs last even after external funding is phased out. Market-based home fortification with micronutrient powder (MNP) was kick started through the efforts of several organizations including Bangladesh Rural Advancement Committee (BRAC) and the Global Alliance for Improved Nutrition (GAIN) in several low- and middle-income countries including in Bangladesh and Kenya (15, 16). However, there is limited evidence on whether market-based MNP interventions implemented nationally have a demonstrated impact on the reduction of anemia among children under age five (15, 16).

From 2014 to 2018, BRAC implemented a market-based home fortification, named the Maternal Infant and Young Child Nutrition (MIYCN) program (17), to reduce anemia among children 6–59 months old. BRAC used an MNP, called Pushtikona-5, which contained five micronutrients (iron, zinc, folic acid, vitamin A, and vitamin C). The micronutrient composition in the Pushtikona-5 includes 12.5 mg of iron (as ferrous fumarate), 5 mg of zinc, 160 μg of folic acid, 300 μg RE of vitamin A, and 30 mg of vitamin C. BRAC’s Shasthya Shebikas (SSs)—female volunteer community health workers—sold MNP sachets to the caregivers of children under age five. The MIYCN program was designed to transform the MNP approach into a well-accepted, large-scale, high-quality, and sustainable intervention, using three mechanisms. First, the program built the capacity of BRAC’s community health workers and incentivized them to increase sales of Pushtikona-5 and promote household compliance with the recommended MNP regimen. Second, the program built stakeholder acceptance of home fortification as a critical component of the MIYCN package and supported policies to enable the distribution of Pushtikona-5 through public and private channels. Third, it created public awareness of and demand for Pushtikona-5 through a mass media campaign. The Pushtikona-5 program was delivered in a phased rollout across three existing program platforms (detailed in Supplementary Table S1) over five years:

1. Phase 1 was delivered to 68 sub-districts in 10 districts and 2 urban slums through the Maternal, Neonatal, and Child Health (MNCH) program platform in October 2014.
2. Phase 2 was delivered to additional 48 sub-districts in 15 districts and 2 urban slums through the Alive & Thrive (A&T) program platform beginning in April 2015.
3. Phase 3 was delivered to additional 48 sub-districts in 9 districts through the BRAC Nutrition program platform in addition to 2 urban slums which began in May 2016.

We evaluated this program concurrently—evaluation activities were carried out alongside the program implementation based on a mixed-methods approach, including qualitative assessments and coverage surveys conducted according to a modified stepped wedged design and including measurement of hemoglobin concentration (17). Several evaluation results have been published elsewhere (18–22). In this paper, we analyze coverage survey data to assess the impact of the program on the prevalence of anemia and hemoglobin concentration among children aged 6–59 months in Bangladesh, on indicators of the coverage of Pushtikona-5, and on the coverage of infant and young child feeding (IYCF) practices. We also consider the outcomes against prevailing secular trends.

## Materials and methods

### Study design

**Figure 1:**
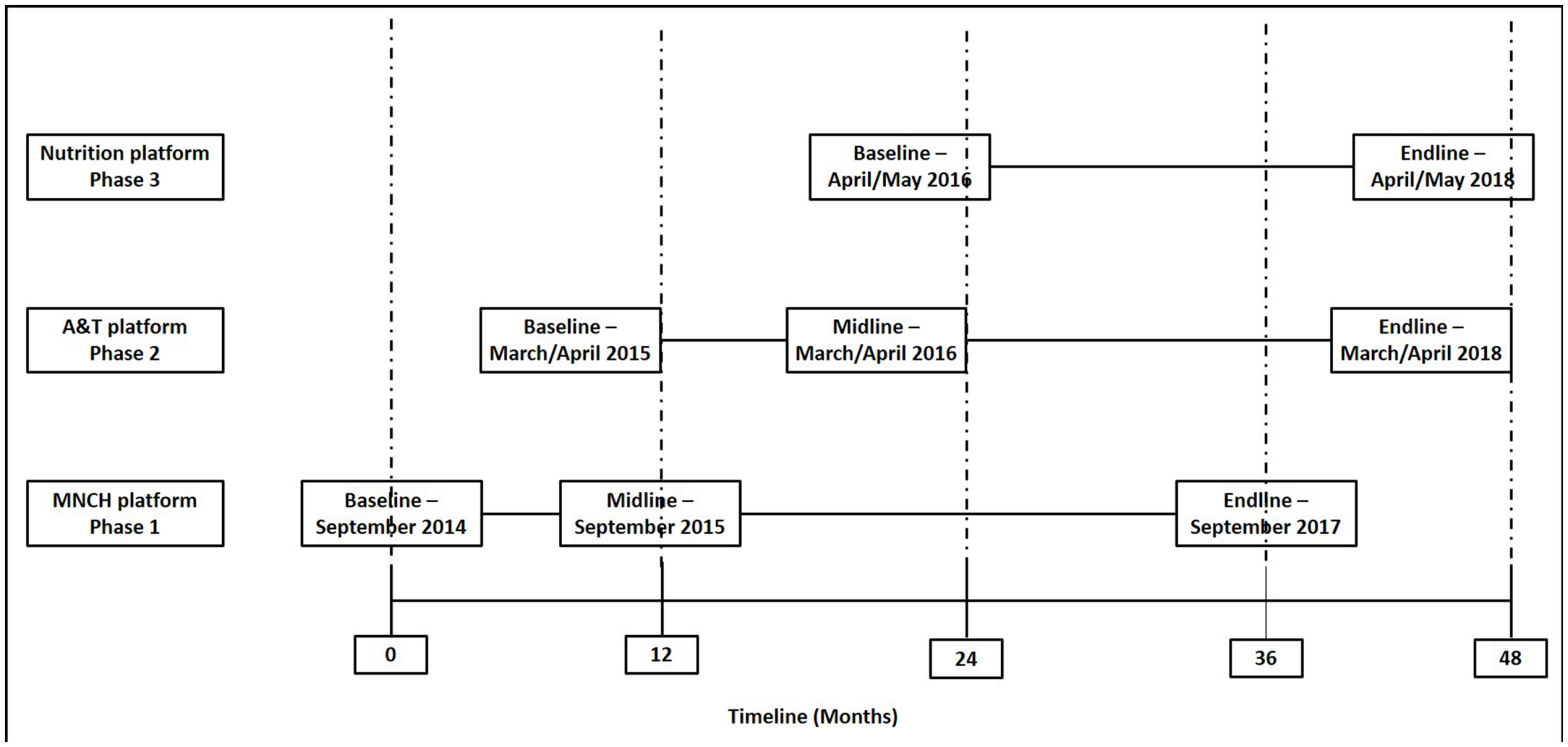
Survey design and timeline of baseline, midline, and endline surveys in three program platforms.

We employed a modified stepped-wedge design to assess the Pushtikona-5 interventions, as the implementation of these interventions commenced at varying times across different program platforms. Several factors informed our choice of this evaluation design. Firstly, it enabled us to evaluate the interventions within the context of routine implementation (23). Secondly, due to resource constraints, it was not feasible to administer interventions simultaneously to all targets (24). Furthermore, this design proved to be pertinent when there was a requirement to detect or control for any time trend effects on the effectiveness of the Pustikona-5 program strategy (25). The program was implemented between September 2014 and May 2018 in the three BRAC program platforms (MNCH, A&T and BRAC Nutrition) in Bangladesh. We conducted three baseline (before implementation), two midline (during implementation), and three endline surveys (post-implementation) to evaluate the outcomes of MIYCN program. All eight surveys were population-based conducted in a cross-sectional nature. Figure 1 describes the evaluation timelines across the three platforms. There were three survey waves in the MNCH and A&T platforms: baseline, midline and endline. However, the BRAC nutrition program undertook only two surveys, one at baseline and another at endline. The study protocol has been prospectively registered with RIDIE (Registry for International Development Impact Evaluation) and registration number is RIDIE-STUDY-ID-553a7c4db7267. The protocol number was PR-14048.

### Participants

The study participants included children aged 6–59 months and caregivers of those children. Children aged 6–59 months were included in the study due to the nutritional transition that occurs after 6 months of age. It is recognized that exclusive breastfeeding is sufficient to meet the nutritional needs of the child before six months. The study centers on a market-based home fortification program with the primary objective of assessing the impact of MNP on anemia. This age group was deliberately chosen as the study population because children within this range are more vulnerable. Understanding the effects of MNP on anemia in this critical developmental stage is of paramount importance. A caregiver was defined as either the child’s biological mother or the person who took care of or looked after the child and gave the child most meals on most days. Households having at least one child aged 6–59 months were selected. The child and the caregiver were eligible to participate if they were not sick on the day of the interview and had no physical or mental challenges that would impede their ability to provide informed consent and respond to questions.

### Sample size estimation

The sample size for the baseline and midline surveys was predicated on estimating coverage of the intervention, which was one of the outcomes of the first phase of the MIYCN program. Limited information was available on coverage of MNP at baseline, because before the implementing the MIYCN program, no large-scale evaluation survey was conducted in Bangladesh; it was therefore assumed to be 50% as this gave the most conservative sample size. At least 192 households, each with one eligible child, would be sufficient to estimate baseline coverage in each district with a precision of 10% of the 95% confidence interval, assuming a design effect of 2.0. A total of 16 clusters were identified from each district and 12 households selected from each cluster.

The sample size calculation for the endline survey was based on detecting a reduction in prevalence of anemia. Using data from the five baseline and midline surveys, the prevalence of anemia was determined to be 42%. In the endline surveys, it was determined that 969 households each with at least 1 eligible child would be sufficient to detect a 10% percentage point decrease in the prevalence of anemia with 90% power at the 5% level of significance assuming a design effect of 2.0 due to clustering. Seven households were selected from each of 22 clusters in each of nine districts included in the endline surveys.

### Selection of study participants

Eligible households were identified using a two-stage cluster sampling procedure. Clusters were defined as the catchment area of a BRAC community health worker, which ranged from 250 to 400 households, with a population ranging from 1,250 to 2,000. At the first stage, the primary sampling units were selected from the list of each district provided by BRAC, using systematic random sampling. For the second stage, we used a physical map segmented sampling and followed WHO’s EPI-5 sampling procedure (26) to identify eligible households. We selected twelve households from each BRAC community in the baseline and midline survey and increased the number of BRAC community along with an additional seven households from each BRAC community in the endline survey. Further details on the sampling process and selection of study participants are published elsewhere (27).

### Data collection

We collected data through interviews with children’s caregivers and collected finger-prick blood samples from the children to measure hemoglobin concentration. For interviews with caregivers, we used a structured questionnaire initially adapted from the WHO’s Food and Nutrition Technical Assistance methodology (28). Trained interviewers administered the questionnaire to the caregivers. The interviewers underwent a comprehensive two-week training period prior to each survey. To gauge their proficiency, a pre-test assessing knowledge of data collection methodologies, study objectives, and research ethics was administered before the final training session. Following the training, a post-test was conducted to further evaluate their performance. Throughout the training, mock interviews were conducted among the interviewers, and constructive feedback was provided to enhance their skills. Additionally, practical sessions were held to practice drawing blood samples. From the pool of trained interviewers, approximately 95% were selected to participate in the final data collection from households. This rigorous training and selection process aimed to ensure the competence and reliability of the interviewers in carrying out the surveys effectively. The trained medical technologists took blood samples from the children by gently pricking either the middle finger or ring finger with a lancet. Using a dry gauze pad, the medical technologist wiped away the first drop of blood to stimulate spontaneous blood flow, gently pressed the finger until the second drop of blood appeared, and ensured that it was big enough to fill the microcuvette completely in one attempt. Hemoglobin concentration was then measured using a HemoCue machine (Hb 301, HemoCue AB, Angelholm, Sweden). We shared the anemia status with the caregiver of the participant child, and any child found to be severely anemic was referred to the nearest health facility (Hb of <7.0 g/dL). A total of 33 severe anemic children were referred to the nearest health facility. This disclosure did not affect the child’s participation in the study.

### Outcomes and covariate measures

The primary outcome was prevalence of anemia defined as hemoglobin concentration less than 11.0 g/dL. Secondary outcomes included infant and young child feeding practices defined aligned with WHO recommendations (28). Coverage levels draw on the Tanahashi model of coverage (29). Message coverage of the home-fortification program was defined as whether the caregiver of a child had heard about MNP. Contact coverage was defined as whether a child had ever consumed food with MNP, and effective coverage, defined as whether a child had consumed three or more sachets of MNP in three or more of the seven days preceding data collection. Total number of MNP sachets consumed by the index child prior to the day of interview were calculated then categorized them into three: none, 1–30 sachets, and > 30 sachets.

The main explanatory variable was survey round as a proxy measure of exposure to the intervention. This given that the intervention to promote home fortification with MNP was rolled out immediately after the baseline survey.

Variables that characterized households, parents and children surveyed include: household size, number of children, income and expenditure; parental age, occupation, level of education and religion; child age, sex and birthweight.

Derived variables included a binary variable derived from total household income to indicate whether it was above or below the median household income and a binary household food security indicator based on the Household Food Insecurity Access Scale (30). Households were then grouped as moderately or severely food insecure for comparison against those classified as food secure or mildly food insecure. Household wealth quintiles were obtained from principal components analysis of indicators of household assets followed by extraction of scores from the first principal component and grouping of these scores into quintiles (31).

### Ethical consideration

This study was conducted according to the guidelines laid down in the Declaration of Helsinki and all procedures involving human subjects/patients were approved by the Institutional Review Board of International Centre for Diarrheal Disease Research, Bangladesh (icddr,b) which consists of two committees, the Research Review Committee and the Ethical Review Committee. Written informed consent was obtained from all subjects/patients’ legal guardians, especially for children.

### Statistical analysis

The proportions of children with anemia, children whose feeding met the criteria of good infant and young child feeding, and of each of the three measured of coverage were calculated at the baseline, midline and endline surveys. Unadjusted risk ratios (RR) comparing these outcomes at midline relative to baseline and endline relative to baseline were estimated using generalized estimating equations with fixed effects for platform and robust standard errors to adjust for clustering of observations within BRAC program districts and survey communities. Risk ratios adjusting for child’s age, child’s sex, household wealth quintile and platform were also estimated. Subgroup analyses exploring the effect of the intervention on anemia and coverage among children who had ever consumed none, up to 30 or more than 30 sachets, and within each of the three platforms, were conducted. Only complete observations were included for all outcomes and covariates. Unadjusted and adjusted RR with 95% confidence intervals were used to present the regression estimates.

## Results

### Sample characteristics

A total of 16,936 households were surveyed. There were 6,892 households in the baseline survey, 4,807 at midline and 5,237 at endline. Table 1 shows the distributions of characteristics of households, parents and children surveyed across the three time-points. Households did not differ significantly with a median size of five members, median household incomes and expenditures of about 90 USD (1 Taka = 0.0089 USD), and a median of one child between the ages of six to 59 months. Household food insecurity varied over time: about a quarter of households were moderately or severely food insecure at baseline (24.8%), rising to about a third at midline (30.2%) then dropping to a fifth at endline (20.4%). Parent and child characteristics did not differ significantly over time. Mothers had a mean age of around 27 years and fathers approximately 33 years. About 76% of mothers reported having at least 5 years of formal education, but only about 64% of fathers reported the same amount of education. A majority, more than 90% of mothers, were housewives, and a similar proportion were Muslims. Fathers earned about 80–90% of the reported household income (Table 1).

**Table 1:** Characteristics of households and children included in the analysis.

Characteristics	Baseline (*N* = 6,892)	Midline (*N* = 4,807)	Endline (*N* = 5,237)	Overall (*N* = 16,936)
Platform				
Maternal, Neonatal and Child Health (MNCH), *n* (%)	1,927 (28.0)	1,924 (40.0)	1,540 (29.4)	5,391 (31.8)
Alive and Thrive Infant and Young Child Feeding (A&T), *n* (%)	2,887 (41.9)	2,883 (60.0)	2,310 (44.1)	8,080 (47.7)
BRAC Nutrition Program (Nutrition), *n* (%)	2,078 (30.2)	0 (0)	1,387 (26.5)	3,465 (20.5)
Household				
Household size, median (range)	5 (2–38)	5 (2–27)	5 (2–25)	5 (2–38)
Household income in Taka, median (range)	10,400 (0–300,000)	10,000 (0–300,000)	14,000 (0–300,000)	12,000 (0–300,000)
Household expenditure in Taka, median (range)	10,000 (0–450,000)	10,000 (0–360,000)	10,000 (1000–150,000)	10,000 (0–450,000)
Household food security, *n* (%)^1^				
Food secure or mildly insecure	3,733 (75.2)	3,356 (69.8)	4,171 (79.6)	11,260 (75.0)
Moderately or severely insecure	1,232 (24.8)	1,451 (30.2)	1,066 (20.4)	3,749 (25.0)
Household wealth quintile, *n* (%)^1^				
1 (lowest)	1,989 (28.9)	844 (17.6)	572 (10.9)	3,405 (20.1)
2	1,259 (18.3)	1,109 (23.1)	1,004 (19.2)	3,372 (19.1)
3	1,244 (18.1)	1,067 (22.2)	1,104 (21.1)	3,415 (20.2)
4	1,169 (17.0)	982 (20.4)	1,213 (23.2)	3,364 (19.9)
5 (highest)	1,231 (17.9)	805 (16.8)	1,344 (25.7)	3,380 (20.0)
Children 6–59 months, median (range)	1 (1–5)	1 (1–6)	1 (1–5)	1 (1–6)
Mother/caregiver				
Age in years, mean (SD)	26.6 (6.0)	27.0 (6.2)	26.5 (5.8)	26.7 (6.0)
Has 5+ years of education, *n* (%)	5,108 (74.1)	3,643 (75.8)	4,186 (79.9)	12,937 (76.4)
Housewife^2^, *n* (%)	1,900 (91.4)	2,725 (94.5)	5,035 (96.1)	9,660 (94.7)
Income in Taka, median (range)	1,200 (0–30,000)	1,000 (0–15,300)	2,000 (0–30,000)	1,500 (0–30,000)
Muslim^2^, *n* (%)	6,241 (90.6)	4,249 (88.4)	4,720 (90.1)	15,210 (89.8)
Father				
Age in years, mean (SD)	33.4 (6.9)	33.9 (7.2)	33.3 (6.5)	33.5 (6.9)
Has 5+ years of education, *n* (%)	4,256 (76.0)	3,057 (69.4)	3,536 (77.3)	10,849 (74.4)
Occupation, *n* (%)				
Unemployed	62 (3.4)	95 (3.5)	213 (4.4)	370 (3.9)
Service sector worker	276 (14.9)	361 (13.4)	662 (13.7)	1,299 (13.8)
Farmer	343 (18.5)	625 (23.1)	983 (20.3)	1,951 (20.8)
Businessman	479 (25.9)	628 (23.2)	1,253 (23.9)	2,360 (25.1)
Migrant worker	23 (1.2)	138 (5.1)	102 (2.1)	263 (2.8)
Day laborer	476 (25.7)	619 (22.9)	1,122 (23.2)	2,217 (23.6)
Other	194 (10.5)	239 (8.8)	498 (10.3)	931 (9.9)
Income in Taka^3^, median (range)	9,000 (0–300,000)	8,000 (0–100,000)	10,000 (0–150,000)	10,000 (0–300,000)
Child				
Age in months, mean (SD)	29.9 (14.7)	30.8 (15.0)	29.0 (14.1)	29.9 (14.6)
Sex, *n* (%)				
Male	3,584 (52.0)	2,523 (52.5)	2,718 (51.9)	8,825 (52.1)
Female	3,308 (48.0)	2,284 (47.5)	2,519 (48.1)	8,111 (47.9)
Reported Birthweight in grams, mean (SD)	2,924 (660.7)	2,952 (642.6)	2,980 (620.6)	2,963 (634.1)

^1^Not included in baseline survey of MNCH platform. ^2^This was the predominant response to this question; all other categories treated as ‘other’. ^3^1 Taka = 0.0089 USD.

### Anemia

The prevalence of anemia was 46.6% at baseline, dropping to 32.1% at midline and 31.2% at endline. The mean hemoglobin concentration was 10.96 g/dL at baseline, increased to 11.31 g/dL at midline and 11.20 g/dL at endline (Table 2). These represented adjusted relative reductions of 34% at midline (RR 0.66, 95%CI 0.62 to 0.71, value of *p* <0.001) and 32% at endline (RR 0.68, 95%CI 0.64 to 0.71, value of *p* <0.001) relative to baseline (Table 2). There was no evidence of a difference in reduction of prevalence of anemia comparing children who had consumed none, up to 30 sachets, or greater than 30 sachets of MNP at midline (interaction *p* value 0.999) or endline (interaction *p* value 0.639) relative to baseline (Table 3). There was also no evidence of a difference in the post-baseline reduction in prevalence of anemia across platforms (interaction *p* values 0.593 and 0.283 for reductions at midline and endline, respectively). There was no evidence of a difference in the post-baseline reduction in prevalence of anemia comparing the surveys conducted in the first year of the program to those conducted between the second and fourth year (interaction *p* value 0.834) (Table 4).

**Table 2:** Effect of the intervention on anemia and MNP coverage.

Outcome	Baseline (*N* = 6,892)	Midline (*N* = 4,807)	Endline (*N* = 5,237)	Risk ratio (95% CI) and value of *p*			
				Midline vs. Baseline		Endline vs. Baseline	
	*n* (%) [x̄]	*n* (%) [x̄]	*n* (%) [x̄]	Unadjusted	Adjusted*	Unadjusted	Adjusted*
Anemia [x̄ hemoglobin g/dL]	3,196 (46.6) [10.96]	1,538 (32.1) [11.31]	1,630 (31.2) [11.20]	0.69 (0.64–0.74) *p* < 0.001	0.66 (0.62–0.71) *p* < 0.001	0.67 (0.64–0.71) *p* < 0.001	0.68 (0.64–0.71) *p* < 0.001
Coverage							
Message coverage – child’s caregiver ever heard about MNP	3,000 (43.5)	3,028 (63.0)	4,746 (90.6)	1.44 (1.37–1.52) *p* < 0.001	1.42 (1.34–1.50) *p* < 0.001	2.07 (1.98–2.16) *p* < 0.001	2.04 (1.95–2.13) *p* < 0.001
Contact coverage – child ever consumed food with MNP	1,671 (24.3)	1,773 (36.9)	3,609 (68.9)	1.51 (1.40–1.64) *p* < 0.001	1.48 (1.37–1.61) *p* < 0.001	2.80 (2.63–2.98) *p* < 0.001	2.76 (2.59–2.94) *p* < 0.001
Effective coverage – consumed 3 or more sachets of MNP in past 7 days	123 (1.8)	217 (4.6)	603 (11.5)	2.51 (1.89–3.34) *p* < 0.001	2.69 (2.03–3.58) *p* < 0.001	6.38 (5.02–8.11) *p* < 0.001	6.15 (4.82–7.85) *p* < 0.001

*Adjusting for child’s age and sex, platform, and household wealth quintile.

**Table 3:** Effect of intervention on anemia and coverage according to number of MNP sachets consumed and within platform.

Outcome	Adjusted* Midline vs. Baseline risk ratio (95% CI)				Adjusted* Endline vs. Baseline risk ratio (95% CI)			
	MNP sachets consumed			Value of *p*	MNP sachets consumed			Value of *p*
	None	1–30	> 30		None	1–30	> 30	
Anemia	0.68 (0.63–0.73)	0.68 (0.55–0.83)	0.68 (0.44–1.06)	0.999	0.76 (0.71–0.81)	0.72 (0.61–0.83)	0.67 (0.46–0.99)	0.639
	Platform			Value of *p*	Platform			Value of *p*
	MNCH	A&T	Nutrition		MNCH	A&T	Nutrition	
Anemia	0.65 (0.59–0.71)	0.67 (0.61–0.74)	_	0.593	0.65 (0.60–0.71)	0.71 (0.65–0.78)	0.64 (0.57–0.72)	0.283
Message coverage	1.34 (1.22–1.47)	1.38 (1.28–1.49)	_	0.597	1.84 (1.71–1.98)	2.02 (1.90–2.16)	2.32 (2.11–2.54)	0.001
Contact coverage	1.36 (1.18–1.56)	1.43 (1.28–1.61)	_	0.553	2.17 (1.94–2.44)	2.83 (2.58–3.11)	3.32 (2.92–3.78)	<0.001
Effective coverage	1.42 (0.90–2.25)	6.25 (3.99–9.81)	_	< 0.001	2.86 (1.87–4.37)	16.61 (10.96–25.18)	4.18 (2.88–6.07)	<0.001

*Adjusting for child’s age and sex, platform, and household wealth quintile. **Not applicable because the definition of subgroups and the outcome are both dependent on MNP awareness or consumption value of ps for test of interaction (difference of effect) by subgroup.

**Table 4:** Comparing post- vs. pre-intervention surveys, adjusting for and investigating interaction with time.*

Outcome	Pre- (*N* = 6,892)	Post- (*N* = 10,044)	Risk ratio (95% CI) and value of *p*			
	*n* (%)	*n* (%)	Unadjusted	Adjusted**	Year 1**	Years 2–4**
Anemia	3,196 (46.6)	3,168 (31.6)	0.77 (0.73–0.82) *p* < 0.001	0.78 (0.73–0.82) *p* < 0.001	_	_
Coverage						
Message coverage – child’s caregiver ever heard about MNP	3,000 (43.5)	7,774 (77.4)	1.66 (1.57–1.74) *p* < 0.001	1.65 (1.57–1.74) *p* < 0.001	1.35 (1.26–1.45)	2.03 (1.85–2.21)
Contact coverage – child ever consumed food with MNP	1,671 (24.3)	5,382 (53.9)	1.92 (1.79–2.06) *p* < 0.001	1.91 (1.78–2.04) *p* < 0.001	1.38 (1.25–1.52)	2.62 (2.30–2.99)
Effective coverage – consumed 3 or more sachets of MNP in past 7 days	123 (1.8)	820 (8.2)	3.01 (2.31–3.91) *p* < 0.001	3.03 (2.33–3.94) *p* < 0.001	_	_

*Binary time variable distinguishing between surveys conducted in the first year versus those in the second through to fourth year. **Adjusting for child’s age and sex, and household wealth quintile. Effects in first vs. later years reported separately where there is evidence of interaction between pre/post-intervention effect and time (value of *p*s < 0.001). No adjustment for platform because there is no data for the nutrition platform in the first year.

### Coverage of micronutrient powder (Pushtikona-5)

At baseline, 43.5% of caregivers surveyed had heard about MNP (message coverage); 24.3% of children had ever consumed food with MNP (contact coverage), and only 1.8% had consumed three or more sachets in the 7 days preceding the survey (effective coverage) (Table 2). These increased to 63.0, 36.9, and 4.6%, respectively, at midline and 90.6, 68.9, and 11.5%, respectively, at endline. The adjusted relative changes were 42% increase in message coverage (RR 1.42, 95%CI 1.34 to 1.50, value of *p* <0.001), 48% increase in contact coverage (RR 1.48, 95%CI 1.37 to 1.61, value of *p* <0.001) and 169% increase effective coverage (RR 2.69, 95%CI 2.03 to 3.58, value of *p* <0.001) at midline and 104% increase in message coverage (RR 2.04, 95%CI 1.95 to 2.13, value of *p* <0.001), 176% increase in contact coverage (RR 2.76, 95%CI 2.59 to 2.94, value of *p* <0.001) and 515% increase in effective coverage (RR 6.15, 95%CI 4.82 to 7.85, value of *p* <0.001) at endline (Table 2).

There was no significant difference in the post-baseline increases in message coverage (interaction *p* value 0.597) or contact coverage (interaction *p* value 0.553) across platforms at midline, but strong evidence of a significant difference in the post-baseline increases effective coverage across platforms at midline (interaction *p* value <0.001) (Table 3). At endline, there was strong evidence of between-platform differences in post-baseline significantly increases in all forms of coverage (all interaction *p* values 0.001 or less).

There was also evidence of a difference in post-baseline increases in message coverage and contact coverage comparing the surveys conducted in the first year of the program to those conducted between the second and fourth year (interaction *p* value <0.001) but no evidence of a difference in the increase in effective coverage over the same time periods (interaction *p* value 0.363) (Table 4).

### Infant and young child feeding practices

There was considerable variation in prevalence of the various components of IYCF practices at baseline (Table 5). Practices such as continued breastfeeding, introduction of complimentary foods, and age-appropriate breastfeeding were highly prevalent, practised by 88% or more of caregivers at baseline. Others, including minimum meal frequency and continued breastfeeding at two years were also highly prevalent at baseline, 80 and 84%, respectively. However, only around half of children received diets of at least the recommended minimum dietary diversity (53%) or minimum acceptable diet (49%).

**Table 5:** Effect of the intervention on IYCF practices.

Outcome	Baseline	Midline	Endline	Risk ratio (95% CI) and value of *p*			
				Midline vs. Baseline		Endline vs. Baseline	
	n/N* (%)	n/N (%)	n/N (%)	Unadjusted	Adjusted**	Unadjusted	Adjusted**
IYCF practices (age of children)							
Continued breastfeeding (12–15 months)	538/580 (92.8)	359/375 (95.7)	440/468 (94.0)	1.03 (1.00–1.06) *p* = 0.037	1.04 (1.01–1.08) *p* = 0.017	1.01 (0.98–1.05) *p* = 450	1.02 (0.99–1.06) *p* = 0.206
Introduction of complimentary foods (6–8 months)	379/408 (92.9)	278/291 (95.5)	240/254 (94.5)	1.03 (0.99–1.07) *p* = 0.133	1.05 (1.01–1.10) *p* = 0.013	1.02 (0.98–1.06) *p* = 0.386	1.03 (0.99–1.06) *p* = 0.163
Minimum dietary diversity (6–23 months)	1,389/2,633 (52.8)	977/1,762 (55.5)	1,134/2,084 (54.4)	1.05 (0.99–1.12) *p* = 0.123	1.04 (0.98–1.11) *p* = 0.195	1.03 (0.97–1.09) *p* = 0.327	0.96 (0.91–1.02) *p* = 0.175
Minimum meal frequency (6–23 months)	2,106/2,622 (80.3)	1,469/1,749 (84.0)	1,583/2,076 (76.3)	1.05 (1.01–1.08) *p* = 0.007	0.99 (0.96–1.03) *p* = 0.654	0.95 (0.92–0.98) *p* = 0.002	0.93 (0.90–0.96) *p* < 0.001
Minimum acceptable diet (6–23 months)	1,272/2,622 (48.5)	908/1,749 (51.9)	995/2,076 (47.9)	1.07 (1.00–1.14) *p* = 0.052	1.02 (0.96–1.09) *p* = 0.519	0.98 (0.92–1.05) *p* = 0.601	0.92 (0.86–0.97) *p* = 0.004
Continued breastfeeding at 2 years (20–23 months)	459/550 (83.5)	348/390 (89.2)	396/461 (85.9)	1.07 (1.02–1.12) *p* = 0.008	1.07 (1.01–1.13) *p* = 0.020	1.03 (0.98–1.09) *p* = 0.244	1.06 (1.00–1.11) *p* = 0.056
Age-appropriate breastfeeding (0–23 months)	2,310/2,622 (88.1)	1,617/1,749 (92.5)	1,894/2,076 (91.2)	1.05 (1.03–1.07) *p* < 0.001	1.06 (1.04–1.09) *p* < 0.001	1.04 (1.01–1.06) *p* = 0.003	1.05 (1.02–1.09) *p* < 0.001

*Denominators depend on number of children in age range. **Adjusting for child’s age and sex, platform, and household wealth quintile.

Feeding practices were mostly static over time with no discernible pattern to any observed changes. There was a 4% adjusted increase in continued breastfeeding at midline relative to baseline (RR 1.04, 95%CI 1.01 to 1.08, value of *p* 0.017) but no adjusted difference at endline (Table 5). Similarly, there was a 5% increase in prevalence of introduction of complimentary foods (RR 1.05, 95%CI 1.01 to 1.10, value of *p* 0.013) but no adjusted difference at endline. The adjusted prevalence of both minimum meal frequency and minimum acceptable diet declined by 7 and 8%, respectively, at endline relative to baseline (RR 0.93, 95%CI 0.90 to 0.96, value of *p* <0.001 and RR 0.92, 95%CI 0.86 to 0.97, value of *p* 0.004 respectively). There was some evidence of increases in the adjusted prevalence of continued breastfeeding at two years (RR 1.07, 95%CI 1.01 to 1.13, value of *p* 0.020 at midline and RR 1.06, 95%CI 1.00 to 1.11, value of *p* 0.056 at endline) and age-appropriate breastfeeding (RR 1.06, 95%CI 1.04 to 1.07, value of *p* <0.001 at midline and RR 1.05, 95%CI 1.02 to 1.09, value of *p* <0.001 at endline).

## Discussion

The results of this study show evidence of a reduction in the prevalence of anemia from baseline to endline and of increased message and contact coverage of Pushtikona-5 from baseline to midline and to endline surveys. However, additional analysis suggested that the observed reduction in anemia prevalence was not due to message and contact coverage and to ever consumption of ≥30 sachets of Pushtikona-5. There was also no evidence of a midline-baseline or endline-baseline difference in the prevalence of anemia according to the effective coverage of Pushtikona-5. This may be because of very low effective coverage of Pushtikona-5 in all platforms in the all three survey time-points.

Given the level of reduction in the prevalence of anemia in the MIYCN program area from baseline to endline, it is possible that the combined interventions (promotion of feeding practice combined with MNP) contributed to the reduction of anemia. Because we did not have an appropriate comparison group, we are unable to attribute the reduced prevalence of anemia in the program districts solely to the interventions. However, home fortification with MNP has shown mixed results in its effects on anemia in previous studies (32–37). Some studies have found that home fortification with MNPs can reduce anemia prevalence and improve hemoglobin levels (32–34). Other studies have found that low-dose iron-containing MNPs did not improve iron status or reduce anemia prevalence (35). The efficacy of MNPs in reducing anemia may depend on factors such as the dose of iron in the MNP, the prevalence of infections, and the frequency of MNP administration (36–38).

There is strong evidence that coverage of Pushtikona-5, particularly message and contact coverage, improved over time. This result suggests that this market-based model can be an appropriate delivery mechanism for home fortification at the community level. In the existing literature, there is limited evidence on the effectiveness of market-based MNP interventions at scale (14, 15, 27); thus this evaluation of a large-scale program using a sales model is a strong addition to the literature. The successful implementation of MNP program may be dependent on the effective operation, quality of training and commitment of community-level health workers. For example, the analyses showed that Pushtikona-5 coverage (including effective coverage) was significantly associated with home visits made by the BRAC community health workers (27). Households of the caregivers who received more visits from the community health workers were more likely to feed Pushtikona-5-fortified foods to their children (27). This finding is consistent with a study conducted in Madagascar (32) showing that interaction with health workers increased consumption of ≥30 sachets of MNP and was associated with a significant reduction in the prevalence of anemia among children. This evaluation also identified a range of individual, household, community, and program-level factors associated with low home visits by the BRAC community health workers, which may be critical for BRAC to consider when implementing similar programs in the future (18, 19, 22).

Our analysis revealed that the prevalence of IYCF practices increased from baseline to midline but remained steady from midline to endline. Several indicators of infant feeding practices were already very high at baseline, potentially leaving little room for further increase. For others, qualitative analysis of this evaluation published elsewhere provides several reasons for low prevalence of other IYCF practices (18, 22). One reason may be that BRAC community health workers were responding to new incentives to promote home fortification with Pushtikona-5, which may have led them to reduce their focus on promoting IYCF (20). When integrating MNP and IYCF interventions, program implementers and stakeholders should be careful to prevent programmatic changes from having unintended negative consequences on program outcomes and should take course-correcting actions as needed during implementation (17, 19). A previous study had recommended that MNP interventions implemented at scale be integrated with IYCF programs because community-based MNP interventions would likely increase community health workers’ contacts with households, thereby strengthening IYCF counseling and support (20, 39, 40).

The evaluation has several limitations and strengths. The evaluation did not have a comparison group, which limited our ability to determine causality regarding the change in prevalence of anemia. The results of the study may not be representative of Bangladesh as a whole or of the districts where the surveys were implemented. We assessed anemia with a drop of blood through capillary finger prick. This process allowed us to measure children’s hemoglobin but limited our ability to measure serum iron and ferritin, which help distinguish the proportion of anemia due to iron deficiency. Single drop capillary hemoglobin assessment is also highly subject to random error (41–43), which may reduce the potential to detect small changes. Additionally, there might have been recall bias, as caregivers may have trouble recalling the use of Pushtikona-5 in the past. Other limitations include the potential for confounding by temporal trends, where comparisons of outcomes between earlier and later periods may be influenced by background changes that affect anemia, irrespective of the program. Furthermore, if temporality was a significant confounder, our sample size might have been compromised by these temporal effects. The Pushtikona-5 program was implemented at large scale in three phases using existing delivery platforms. The use of a stepped wedged survey design, and collected data from all program districts is an important strength, ensuring feasibility for evaluation in real-world program settings (18).

## Conclusion

These results show evidence of a reduction in the prevalence of anemia and an improvement in Pushtikona coverage and some infant and young child feeding practices across the three surveys. However, we found no evidence that any observed improvements in anemia were different from the prevailing secular trends. Results for message and contact coverage for which improvements were greater in the latter years than the initial years of the program suggest positive effects of the intervention. This study provides important evidence of the feasibility and potential for impact of linking market-based MNP distribution with IYCF promotion through community level health workers. Anemia reduction however, will likely require additional actions that address the multi-causality of the condition.

## Data availability statement

The raw data supporting the conclusions of this article will be made available by the authors, without undue reservation.

## Ethics statement

The studies involving humans were approved by the Institutional Review Board of icddr,b, which consists of two committees, the Research Review Committee and the Ethical Review Committee. The studies were conducted in accordance with the local legislation and institutional requirements. Written informed consent for participation in this study was provided by the participants’ legal guardians/next of kin.

## Author contributions

HS: Conceptualization, Data curation, Formal analysis, Funding acquisition, Investigation, Methodology, Project administration, Resources, Software, Supervision, Validation, Visualization, Writing – original draft, Writing – review & editing. MR: Data curation, Methodology, Writing – review & editing. MT: Methodology, Writing – review & editing, Conceptualization, Formal analysis, Funding acquisition. MI: Methodology, Writing – review & editing, Data curation, Investigation, Project administration. MM: Conceptualization, Methodology, Writing – review & editing. GA: Conceptualization, Writing – review & editing, Methodology. SA: Conceptualization, Funding acquisition, Writing – review & editing. CH: Conceptualization, Methodology, Writing – review & editing, Funding acquisition. RK: Methodology, Writing – review & editing, Conceptualization. MB: Data curation, Methodology, Writing – review & editing. SS: Data curation, Writing – review & editing, Methodology. MAR: Data curation, Methodology, Writing – review & editing. SAS: Writing – review & editing, Data curation. MC: Writing – review & editing. KA: Writing – review & editing, Conceptualization. SG: Writing – review & editing. CB: Formal analysis, Methodology, Writing – review & editing, Data curation. CD’E: Formal analysis, Methodology, Writing – review & editing. MS: Conceptualization, Formal analysis, Funding acquisition, Methodology, Writing – review & editing, Investigation. LN: Conceptualization, Formal analysis, Funding acquisition, Methodology, Writing – review & editing. TA: Conceptualization, Data curation, Formal analysis, Funding acquisition, Investigation, Methodology, Project administration, Supervision, Writing – review & editing.
