# Supplementary material for: Impact of market-based home fortification with micronutrient powder on childhood anemia in Bangladesh: a modified stepped wedge design
Source: Front Nutr. 2024 Jan 5;10:1271931. doi: 10.3389/fnut.2023.1271931 (PMC10796820; doi:10.3389/fnut.2023.1271931)
Supplement: Supplementary file 1 [file Table_1.DOCX]

**Table S1: BRAC’s existing interventions implemented in three program platforms and additional home fortification interventions in all three platforms**

| **BRAC program platform** | **Existing interventions under each platform** | **Additional interventions supported under all three platforms** |
| --- | --- | --- |
| Maternal, Neonatal, and Child Health (MNCH) | - Provision of basic primary healthcare at the community level - Work with village health committees to motivate behavior change in the community by addressing pregnancy and newborn and child health - Facilitation of access to obstetric and newborn care at public and private health facilities | - Training (a basic training workshop and monthly refresher training) to BRAC’s CHWs to promote home fortification with MNP at the household level - Sale of MNPs and provision of counseling to the caregivers of children aged 6–59 months on home fortification with MNPs, monitoring of home fortification activities at the community level by CHWs, and the provision of incentives to CHWs to promote MNP at the community level - Demand-generation activities included social and behavior change communication through various channels (i.e., mass media, social mobilization) to sensitize the community and gatekeepers to increase demand for MNPs - Interventions for creating enabling an environment for home fortification with MNPs included advocacy to national-level key stakeholders toward enabling policy, building of government leadership and buy-in on home fortification with MNPs, and efforts to ensure that key stakeholders and civil society support and demand home fortification as part of optimum IYCF practices |
| Alive and Thrive (A&T) | - Home visits by BRAC community health workers (CHWs) to provide infant and young child feeding (IYCF) counseling, coaching, demonstration, and problem solving - Antenatal care sessions and postnatal care visits by the CHWs for early initiation of breastfeeding, exclusive breastfeeding, and support for good positioning and attachment - Health forums where the CHWs disseminate IYCF messages and discuss issues in small groups with pregnant women, mothers, and family members |  |
| Nutrition | - BRAC CHWs visit households in their communities and provide counseling, coaching, and demonstrations to raise awareness on nutrition - CHWs offer community-based management of acute malnutrition (CMAM) services and provide supplementary food to mothers and children aged 6–59 months affected by moderate acute malnutrition - CHWs raise awareness about adolescent girls’ nutrition and encourage mothers and family members about issues like intake of healthy food and good IYCF practices |  |
