# Supplementary material for: Impact of market-based home fortification with micronutrient powder on childhood anemia in Bangladesh: a modified stepped wedge design
Source: Front Nutr. 2024 Jan 5;10:1271931. doi: 10.3389/fnut.2023.1271931 (PMC10796820; doi:10.3389/fnut.2023.1271931)
Supplement: Supplementary file 2 [file Table_2.DOCX]

**Table S2: Descriptions of variables including type of variables and their definitions**

| **Variable** | **Type** | **Definition** |
| --- | --- | --- |
| Household size | Continuous, we categorized (<5, ≥5) based on  median household size | Number of members in a household |
| Caregiver’s education | Categorical (<5 years of schooling, ≥5 years of schooling) | Caregiver’s years of schooling |
| Caregiver’s age | Continuous | Age of the caregiver of the index child |
| Caregiver’s religion | Categorical (Hindu/Muslim/Christian/  Buddhist/Other) | Religious status of the caregiver of the index child |
| Father’s education | Categorical (<5 years of schooling, ≥5 years of schooling) | Father’s (index child’s father) years of schooling |
| Father’s age | Continuous | Age of father of the index child. |
| No. of children aged  6–59 months | Continuous | Total number of children aged 6–59 months in a household |
| Child’s sex | Categorical (male/female) | Sex of the index child |
| Message coverage | Categorical (yes/no) | Caregivers who had heard of MNPs in the week prior to the day of survey |
| Contact coverage | Categorical (yes/no) | Caregivers who had ever fed MNPs to child |
| Effective coverage | Categorical (yes/no) | Caregivers who had fed at least 3 sachets of MNPs to child in the week prior to the day of interview |
| Consumption of MNP | Categorical (none, 1-30 sachets, >30 sachets) | Total number of MNP sachets consumed by the index child prior to the day of interview |
| Wealth quintile | Categorical (poorest, poorer, middle, richer, richest) | Wealth index was measured by assets of the households by principal component analysis |
| Anemia | Categorical (anemic/non-anemic) | Child whose hemoglobin level was <11.0 g/dl was defined as anemic |
| Hemoglobin concentration | Continuous | Hemoglobin level of blood for under-five children |
| Food security status of household | Categorical (food secure or mildly, moderately, or severely food insecure) | Food security status of household was calculated based on the household food insecurity access scale |
| Household income | Continuous | Income of the household members who earned |
| Birth weight | Continuous | Birth weight of the child was recorded from birth registration cards or from recall |
| Continued breastfeeding | Categorical (yes/ no) | Children aged 12-15 continued breastfeeding |
| Introduction of complementary foods | Categorical (yes/ no) | Children introduced solid semi-solid or soft foods at 6-8 months of their age |
| Minimum dietary diversity | Categorical (yes/ no) | Children fed foods from diversified groups |
| Minimum meal frequency | Categorical (yes/ no) | Children fed foods at least minimum number of times |
| Minimum acceptable diet | Categorical (yes/ no) | Children met the criteria of both minimum dietary diversity and minimum meal frequency |
| Continued breastfeeding at 2 years | Categorical (yes/ no) | Children aged 20-23 months of age continued breastfed |
| Age-appropriate breastfeeding | Categorical (yes/ no) | Children aged 0-23 months of age continued breastfed |
